# Pavlovian-conditioned alcohol-seeking behavior in rats is invigorated by the interaction between discrete and contextual alcohol cues: implications for relapse

**DOI:** 10.1002/brb3.216

**Published:** 2014-02-06

**Authors:** Jessica Remedios, Catherine Woods, Catherine Tardif, Patricia H Janak, Nadia Chaudhri

**Affiliations:** 1 Center for Studies in Behavioural Neurobiology/Groupe de recherche en neurobiologie comportementale, Department of Psychology, Concordia UniversityMontreal, Quebec, Canada; 2 Center for Neural Science, New York UniversityNew York, New York; 3 Ernest Gallo Clinic & Research Center, University of California at San FranciscoEmeryville, California; 4 Department of Neurology, University of CaliforniaSan Francisco, California; 5 Wheeler Center for the Neurobiology of Addiction, University of California at San FranciscoSan Francisco, California

**Keywords:** Alcoholism, context, ethanol, learning, Pavlovian-conditioning, reinstatement, renewal

## Abstract

**Introduction:**

Drug craving can be independently stimulated by cues that are directly associated with drug intake (discrete drug cues), as well as by environmental contexts in which drug use occurs (contextual drug cues). We tested the hypothesis that the context in which a discrete alcohol-predictive cue is experienced can influence how robustly that cue stimulates alcohol-seeking behavior.

**Methods:**

Male, Long-Evans rats received Pavlovian discrimination training (PDT) sessions in which one conditioned stimulus (CS+; 16 trials/session) was paired with ethanol (0.2 mL/CS+) and a second stimulus (CS−; 16 trials/session) was not. PDT occurred in a specific context, and entries into a fluid port where ethanol was delivered were measured during each CS. Next, rats were acclimated to an alternate (nonalcohol) context where cues and ethanol were withheld. Responses to the nonextinguished CS+ and CS− were then tested without ethanol in the alcohol-associated PDT context, the nonalcohol context or a third, novel context.

**Results:**

Across PDT the CS+ elicited more port entries than the CS−, indicative of Pavlovian discrimination learning. At test, the CS+ elicited more port entries than the CS− in all three contexts: however, alcohol seeking driven by the CS+ was more robust in the alcohol-associated context. In a separate experiment, extinguishing the context-alcohol association did not influence subsequent CS+ responding but reduced alcohol seeking during non-CS+ intervals during a spontaneous recovery test.

**Conclusion:**

These results indicate that alcohol-seeking behavior driven by a discrete Pavlovian alcohol cue is strongly invigorated by an alcohol context, and suggest that contexts may function as excitatory Pavlovian conditioned stimuli that directly trigger alcohol-seeking behavior.

## Introduction

Craving, a term that characterizes an addict's subjective urge to consume a drug, is a central factor associated with relapse (Sinha and O'Malley 1999; Litt et al. 2000; Flannery et al. 2001; Evren et al. 2010). Craving can be triggered by environmental stimuli that, through repeated co-occurrence with drugs of abuse, can come to predict the pharmacological effects of addictive substances (Ludwig 1986; Field and Duka 2002; Uslaner et al. 2006). For example, the sensory properties of alcohol (e.g., sight, smell, taste) evoke craving and physiological reactivity in individuals with alcohol abuse disorders, which may in turn promote drinking (Ludwig and Wikler 1974; Pomerleau et al. 1983; Litt and Cooney 1999).

The sensory properties of an orally consumed drug like alcohol are typically encountered as temporally “discrete” events because they gain prominence while the drug is actively being consumed. Conversely, specific configurations of multi-modal environmental stimuli that are present in the background during drug use do not necessarily gain or lose prominence in relation to drug intake. Nonetheless, like discrete cues, environmental contexts evoke craving in humans (Bordnick et al. 2008; Conklin et al. 2008, 2010; Paris et al. 2011) and drug seeking in animals (Crombag and Shaham 2002; Zironi et al. 2006; Fuchs et al. 2007; Chaudhri et al. 2008b; Perry and McNally 2013), suggesting that they too acquire the capacity to predict drug availability through Pavlovian learning (Janak and Chaudhri 2010). Determining how discrete and contextual drug cues independently influence relapse has been a long-standing empirical question. However, given that these two types of environmental stimuli frequently co-occur in the everyday experience of drug users, it is of value to understand the impact that their co-occurrence may have on craving and drug seeking (Litt and Cooney 1999; Paris et al. 2011; Nees et al. 2012).

We investigated this question using a behavioral animal model of Pavlovian-conditioned alcohol seeking in which rats were trained in a specific context to discriminate between two auditory conditioned stimuli (CS), a CS+ that was paired with alcohol and a CS− that was presented without alcohol (Chaudhri et al. 2008b, 2010). Entries into the fluid port where alcohol was delivered for oral consumption were measured during both cues to assess discrimination. Following training, rats were repeatedly exposed to a different context where neither the cues nor alcohol were presented, in order to acclimate them to an environment that was never associated with alcohol availability or consumption. At test, Pavlovian-conditioned alcohol seeking was measured by presenting the non-extinguished CS+ and CS− without alcohol in the alcohol-associated context, the nonalcohol context, or a novel context. In a separate experiment, we sought to determine if the impact of the alcohol-associated context on responding elicited by the CS+ was mediated by the capacity of the alcohol context to function as an excitatory Pavlovian-conditioned stimulus. We predicted that repeated exposure to the alcohol context after Pavlovian-conditioning would extinguish the association between the context and alcohol, and result in reduced responding to the CS+ at test relative to rats that did not receive context extinction.

## Materials and Methods

### Subjects

Subjects were male, Long-Evans rats weighing 220–240 g on arrival (Experiment 1: Charles River, St-Constant, Québec, Canada; Experiments 2 and 3: Harlan, Indianapolis, IN). Rats were individually housed in temperature (20 ± 1°C) and humidity-controlled colony rooms on a 12-h light/dark cycle (lights on at 7:00 am) with experimental procedures conducted during the light phase. They had unrestricted access to standard rat chow and water throughout the experiments (except as described below), and were weighed and handled in the colony room daily during an initial 7-day acclimation period. The Institutional Animal Care and Use Committee at the Ernest Gallo Clinic and Research Center and the Animal Research Ethics Committee at Concordia University approved all experimental procedures, which are in agreement with recommendations in the *Guide for the Care and Use of Laboratory Animals* (Institute of Laboratory Animal Resources, Commission of Life Sciences, National Research Council, 1996).

### Apparatus

Behavioral procedures were conducted using equipment and software from Med Associates Inc. (St. Albans, VT). Operant conditioning chambers (ENV-009A, 30.5 cm L × 31.8 cm W × 29.2 cm H) were contained within ventilated sound-attenuating cubicles (70–75 dB background noise). Chambers consisted of clear Plexiglas front-doors, ceilings and back-walls, aluminium side walls, and floors made of stainless steel bars. A white house light (ENV-215M, 2.8 W) was located centrally, near the ceiling on the left wall, next to a white noise generator (ENV-225SM, 80–85 dB) and clicker stimulus (ENV-135M, 80–85 dB). The right wall contained a fluid delivery receptacle (referred to as a port) located 2 cm above the floor (ENV-200R3AM) that was connected to a 20-mL syringe via polyethylene tubing. The syringe was placed on a syringe pump (PHM-100, speed 3.33 RPM) outside the sound-attenuating cubicle. Entries into the fluid port were measured by interruption of an infrared beam across its entrance. A PC computer, running Med PC IV software, controlled presentations of the auditory stimuli and pump activation, and recorded entries into the fluid port.

### Drugs

Ethanol (EtOH) solutions were prepared by combining 95% ethanol in tap water to obtain 15% EtOH (v/v; Experiment 1) or 20% EtOH (v/v; Experiments 2 and 3). Sweetened EtOH was prepared by combining 95% ethanol and sucrose in tap water to obtain either a 2% sucrose–15% EtOH (w/v; Experiment 1) or a 2% sucrose–20% EtOH (w/v; Experiments 2 and 3).

### Alcohol exposure in the home cage

Rats were initially acclimated to the taste and pharmacological effects of EtOH in the home cage. This procedure was the same for Experiments 2 and 3, but differed for Experiment 1.

In Experiment 1, rats (*n* = 25) first received a 24-h session in which only 15% EtOH was available in the home cage, followed by a 24-h session in which only water was available. Subsequently, they received 15% EtOH for 1 h/day (during the light phase) and water for 23 h/day for 18 consecutive days. EtOH was restricted to 1 h to encourage consumption within a time frame that corresponded to the length of subsequent behavioral sessions.

Experiment 2 (*n* = 32) and Experiment 3 (*n* = 28) utilized an intermittent, 24-h access, two-bottle choice procedure that produces high EtOH intakes in outbred rats (Wise 1973; Simms et al. 2008; Sparks et al. 2013). On Monday, Wednesday and Friday rats received concurrent access to one bottle containing water and a second bottle containing 20% EtOH for 24-h sessions across 5–6 weeks. On Tuesday, Thursday, Saturday and Sunday only water was available.

In all experiments, the left/right positions of water and EtOH bottles were alternated daily to mitigate the impact of side preferences. Rat weights and volume of ethanol consumed was obtained for each session and used to calculate EtOH intake in terms of g/kg (grams of EtOH consumed divided by rat weight in kilograms). Spillage was accounted for by subtracting the volume of fluid lost from bottles on an empty cage. Rats that consumed less than 1.0 g/kg by session 7 were given sweetened EtOH for 2–3 sessions to entice drinking. Rats with the highest EtOH intakes averaged across the last 2 days (Table 1) were selected for behavioral testing.

**Table 1:** Ethanol intake averaged over the last two sessions (mean ± SEM) of exposure in the home cage or Pavlovian discrimination training.

	Ethanol concentration (vol/vol)	Home-cage ethanol consumption (g/kg)	Pavlovian discrimination training (g/kg)
Experiment 1 (*n* = 16)	15%	0.47 ± 0.05	0.70 ± 0.03
Experiment 2 (*n* = 26)	20%	3.79 ± 0.24	1.02 ± 0.01
Experiment 3 (*n* = 18)	20%	4.10 ± 0.03	1.11 ± 0.02

Values represent grams of ethanol consumed as a function of body weight in kilograms (g/kg).

### Pavlovian discrimination training

Pavlovian discrimination training (PDT) was conducted in daily, 60-min sessions, Monday–Friday. At 5 min after placement into the operant conditioning chamber the house light was illuminated to indicate the start of the session. In each session, rats received 16 presentations each of two different 10-sec auditory conditional stimuli (CS), a continuous white noise and clicker (2 Hz), controlled by a variable-time 67-sec schedule. Presentations of one stimulus (CS+) were paired with EtOH (concentration as per experiment), whereas presentations of the second stimulus (CS−) were not. EtOH (0.2 mL/CS+; 3.2 mL/session) was delivered into the fluid port for oral consumption over the last 6 sec of each CS+. Ports were checked at the end of each session to ensure that all the EtOH had been consumed. Ethanol intake in g/kg averaged across the last two sessions of this phase for each experiment is reported in Table 1.

PDT was conducted in a context that remained constant for each rat (see Table 2 for configuration of contexts utilized in each experiment). Rats were counterbalanced into their assigned PDT context based on EtOH intake at the end of home-cage exposure. The assignment of either the white noise or clicker as the CS+ was counterbalanced across context and kept constant for each rat. Before PDT began rats were habituated to each context in single 20-min sessions without any auditory cues or EtOH.

**Table 2:** Configuration of contextual stimuli that comprised each context type.

Experiment	Context type	Tactile stimulus (floor)	Visual stimulus (walls)	Olfactory stimulus
1	1	Smooth Plexiglas	Black	Lemon
	2	Wire mesh	Clear Plexiglas	Almond
2	1	Wire mesh	Clear Plexiglas	Strawberry
	2	Smooth Plexiglas	Black	Lemon
	3	Bar floor	Black and white vertical stripes	Almond
3	1	Smooth Plexiglas	Black	Lemon
	2	Wire mesh	Clear Plexiglas	Almond

Inserts were placed over the floors of the operant chambers to create smooth Plexiglas or wire mesh tactile stimuli. Walls were covered with paper to create black or black and white striped visual stimuli. Drops (3–4) of lemon or almond extract were placed inside the waste pan or a strawberry air freshener was taped outside the door to serve as olfactory stimuli.

### Exposure to an alternate, nonalcohol context

At 24 h after the last PDT session rats received sessions in which they were exposed to a context (referred to as the nonalcohol context) that differed from the PDT environment. During each 60-min session the house light was illuminated, but auditory cues were withheld and EtOH was not delivered. Empty syringe pumps continued to be activated on a variable-time 67-sec schedule.

### Test

At test, responding to the CS+ and CS− was evaluated by presenting both cues as they occurred during PDT, but without EtOH. The empty syringe pump was activated during the CS+, but no EtOH was delivered. Responding to the cues was assessed under several different conditions, explained in detail below.

### Experiment 1: Pavlovian-conditioned alcohol seeking in an alcohol-associated context or nonalcohol context

Using 18 rats with the highest EtOH intakes during home-cage exposure, we tested the hypothesis that the context in which a discrete alcohol-predictive cue is encountered determines how vigorously that cue triggers conditioned alcohol seeking. Two rats were excluded following behavioral training because they failed to acquire conditioned responding to the CS+. Rats received 14 PDT sessions (final sample sizes: Context Type 1, *n* = 9; Context Type 2, *n* = 7) where the CS+ was paired with 15% EtOH, followed by eight sessions of alternate-context exposure. Subsequently, responding to the CS+ and CS− was assessed in two tests administered 24-h apart. For half the rats, test 1 was conducted in the alcohol-associated PDT context, whereas for the remaining rats it was conducted in the nonalcohol context. The context was then reversed at test 2, according to a counterbalanced, within-subjects design.

### Experiment 2: Pavlovian-conditioned alcohol seeking in an alcohol-associated context, nonalcohol context or novel context

Here we investigated the possibility that removal from the alternate, nonalcohol context was sufficient to elevate CS+ responding at test. Rats (*n* = 32) received 19 PDT sessions where the CS+ was paired with 20% EtOH. Six rats were subsequently dropped because they failed to acquire robust discrimination (final sample sizes: Context Type 1, *n* = 10; Context Type 2, *n* = 8; Context Type 3, *n* = 8). Next, six sessions of exposure to the alternate, nonalcohol context were conducted, counterbalanced across the two context-types that were not utilized during PDT. At test, responding to the CS+ and CS− without EtOH was assessed in the alcohol-associated PDT context, the nonalcohol context or a novel context. The novel context consisted of the remaining context type that had not been utilized during either PDT or alternate, nonalcohol context exposure. Each rat was tested in each of the three conditions using a within-subjects design, with one PDT session and three sessions of alternate-context exposure between tests. These additional sessions were conducted in order to minimize response decrements produced by experiencing the CS+ without ethanol at test.

### Experiment 3: Impact of context extinction on Pavlovian-conditioned alcohol-seeking behavior

This study investigated if the impact of the alcohol context on responding elicited by the CS+ was mediated by the capacity of the alcohol context to function as an excitatory Pavlovian-conditioned stimulus. We predicted that extinguishing the context-alcohol association after PDT would diminish the influence of context on CS+ responding when both cues were subsequently tested in PDT context. Rats (*n* = 24) received 15 PDT sessions where the CS+ was paired with 20% EtOH. Six rats were dropped because they failed to acquire PDT (final sample sizes: Context Type 1, *n* = 8; Context Type 2, *n* = 10). The remainder received eight sessions of exposure to the alcohol-associated PDT context (Group 1; *n* = 9) or to an alternate context (Group 2; *n* = 9). In both cases, the cues and EtOH were withheld. At test, the CS+ and CS− were presented without alcohol in the PDT context (Test 1). A second, identical test was conducted 10 days later to determine the impact of context extinction on spontaneous recovery of CS+ responding (spontaneous recovery test). Between tests, rats remained in their home cages and were handled regularly.

### Statistical analyses

Dependent variables for PDT and test included: normalized CS+ and normalized CS− responding (calculated by subtracting port entries during 10-sec intervals before each CS from port entries during the corresponding CS); post-CS+ responding (port entries during 10-sec intervals after each CS+); total port entries (number of port entries per session); and responding outside CS+ (total port entries minus CS+ responding). The number of port entries made during each CS+ trial at test was analyzed in blocks of two trials, yielding a total of 8 blocks. During exposure to the alternate-context or context-extinction sessions only total port entries were recorded.

PDT data were analyzed using analysis of variance (ANOVA) with CS (CS+, CS−) and Session (as per number of sessions) as within-subject repeated-measures. Total port entries across PDT and total port entries during alternate-context exposure or context extinction were analyzed separately across the within-subject factor of Session (as per number of sessions). Test data for Experiments 1 and 2 were analyzed with CS (CS+, CS−) and Test Context as within-subject repeated-measures. For Experiment 3, data from Test 1 and the spontaneous recovery test were analyzed separately across the between- and within-subject factors of Group (context-extinction; alternate-context) and CS (CS+, CS−), respectively. Port entries averaged across blocks of two CS+ trials at test were analyzed across the within-subject factors of Block (1–8) and Test Context for Experiment 1, and Block (1–8) and Group (context-extinction, alternate-context) for Experiment 3.

Mauchly's Test of Sphericity was used to examine homoscedasticity and the Huynh-Feldt correction was applied when data violated the assumption of sphericity. Statistically significant main effects and interactions were investigated using *t*-tests for paired- or independent-samples. The criterion for statistical significance was *P* = 0.05. Analyses were conducted using SPSS v 11 (Chicago, IL).

## Results

### Experiment 1: Pavlovian-conditioned alcohol seeking in an alcohol-associated context or nonalcohol context

**Figure 1:**
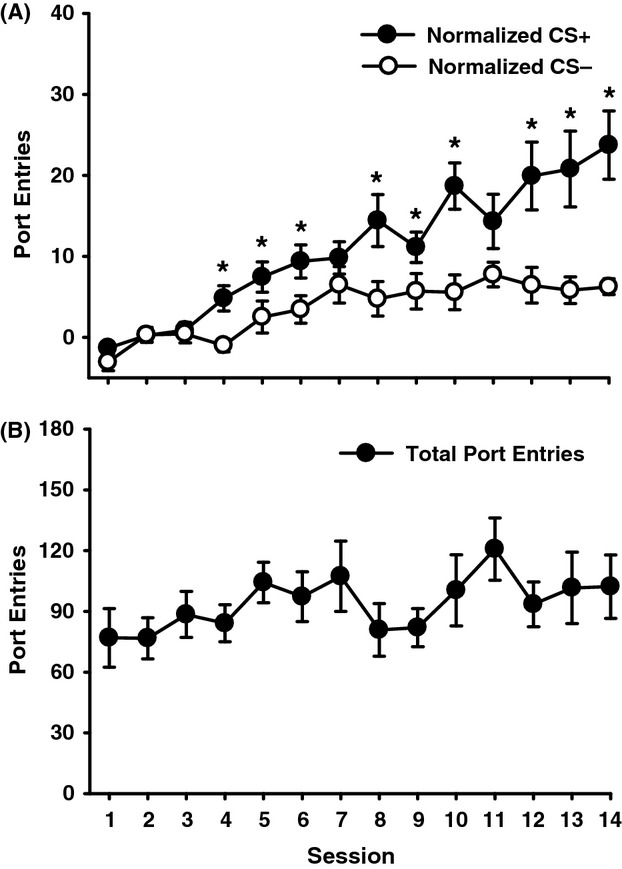
Acquisition of Pavlovian discrimination training for 16 rats across 14 sessions where each CS+ trial was paired with 0.2 mL of 15% ethanol. CS− trials were not paired with ethanol. (A) Mean (± SEM) normalized port entries during the CS+ (filled circles) and CS− (open circles). Normalized data were obtained by subtracting pre-CS responding from responding during the corresponding CS. (B) Mean (± SEM) total number of port entries made during each PDT session. *P < 0.05, CS+ versus CS−.

Rats learned to discriminate between the alcohol-paired CS+ and the CS− (Fig. 1A). Normalized port entries during the CS+ increased across session, whereas CS− responding stabilized at a lower level (Session, *F*(13, 195) = 10.50, *P* < 0.001; CS, *F*(1, 15) = 31.56, *P* < 0.001; Session × CS, *F*(13, 195) = 5.92, *P* < 0.001). The number of total port entries per session (Fig. 1B) remained stable across PDT (Session, *F*(13, 195) = 1.25, *P* = 0.28).

**Figure 2:**
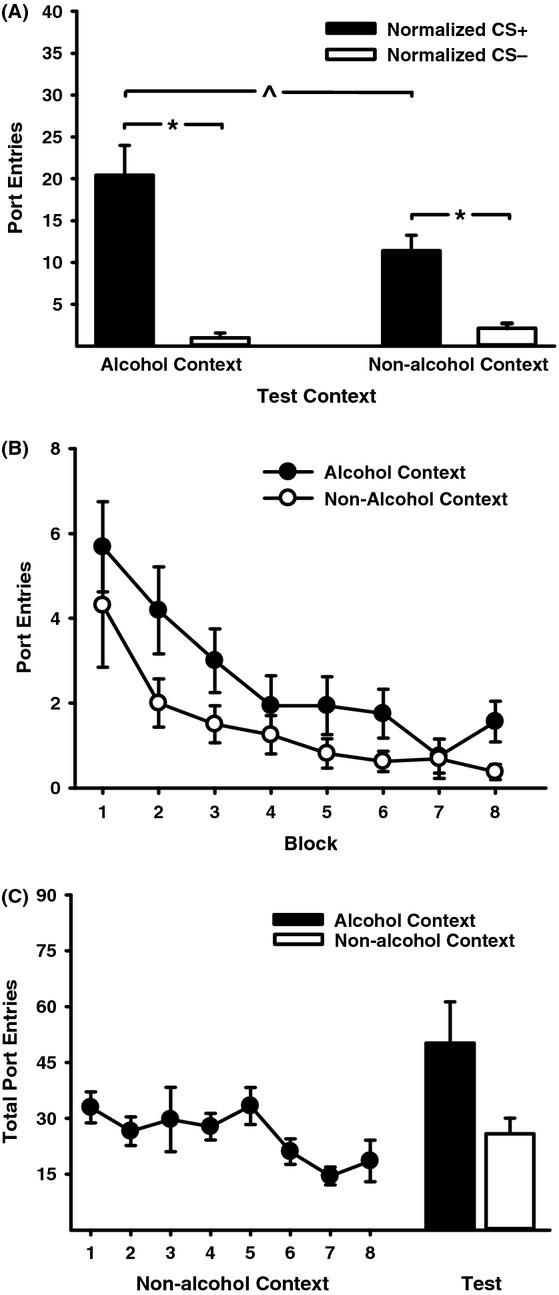
Responding to an alcohol-predictive CS+ is invigorated in an alcohol context, compared to a nonalcohol context. (A) Mean (± SEM) normalized port entries during the CS+ (filled bars) and CS− (open bars) at test in the alcohol context and nonalcohol context. At test the CS+ and CS− were presented without ethanol. (B) Mean (± SEM) port entries averaged across blocks of two CS+ trials at test in the alcohol context (filled circles) and nonalcohol context (open circles). (C) Mean (± SEM) total port entries across sessions of exposure to the alternate (nonalcohol) context (filled circles) in which neither cues nor alcohol were presented. Bars represent mean (± SEM) total port entries at test in the alcohol context (filled bar) and nonalcohol context (open bar). *, ^*P* < 0.05 for indicated comparisons.

At test, the number of port entries triggered by the CS+ was significantly higher in the alcohol-associated context, than in the nonalcohol context (Fig. 2). ANOVA conducted on normalized CS responding (Fig. 2A) revealed a significant main effect of CS (*F*(1, 15) = 46.90, *P* < 0.001), and follow-up *t*-tests for paired-samples verified a significant difference responding to the CS+ and the CS− in the alcohol context (*t*(15) = 5.70, *P* < 0.001) and nonalcohol context (*t*(15) = 4.86, *P* < 0.001). There was a near significant main effect of Test Context (*F*(1, 15) = 3.81, *P* = 0.07) and a significant Test Context × CS interaction (*F*(1, 15) = 7.98, *P* = 0.01). Paired-samples *t*-tests revealed that CS+ responding was higher in the alcohol context than in the nonalcohol context (*t*(15) = 2.41, *P* = 0.03). There was no statistically significant difference across context in responding to the CS− (*t*(15) = −1.42, *P* = 0.18). Port entries made during consecutive CS+ trials (Fig. 2B) decreased across the test (Block, *F*(7, 105) = 4.74, *P* = 0.003), with a near significant Block × Test Context interaction (*F*(7, 105) = 2.26, *P* = 0.07). Overall, responding in the alcohol context was elevated when compared to the nonalcohol context (Test Context, *F*(1, 15) = 15.32, *P* = 0.001). Paired-samples *t*-tests conducted to investigate the specific prediction that context influences CS+ responding on a trial-by-trial basis revealed that in block 8 responding to the CS+ was significantly higher in the alcohol context, than in the nonalcohol context (*t*(15) = 2.33, *P* = 0.03). Figure 2C depicts total port entries during sessions of exposure to the nonalcohol context that followed PDT, as well as total port entries obtained at test. Repeated exposure to the nonalcohol context without cues or alcohol caused a decreasing trend in total port entries across sessions (Session, *F*(7, 105) = 2.32, *P* = 0.08). At test, the total number of port entries was significantly higher in the alcohol context, compared to the nonalcohol context (Test Context, *F*(1, 15) = 5.32, *P* = 0.04). When the number of port entries made during CS+ trials was subtracted from total port entries to estimate alcohol-seeking behavior that was not signalled by the CS+ at test, data indicated a trend (*t*(15) = 1.87, *P* = 0.08) for more port entries to be made outside the CS+ in the alcohol context (mean = 29.56, SEM ± 9.08) than in the nonalcohol context (mean = 14.25, SEM ± 3.10). There was no impact of context on port entries made during the 10-sec post-CS+ interval (*t*(15) = 7.01, *P* = 0.49). Thus, the alcohol context caused a selective increase in alcohol-seeking behavior driven by the CS+.

### Experiment 2: Pavlovian-conditioned alcohol seeking in an alcohol-associated context, nonalcohol context or novel context

**Figure 3:**
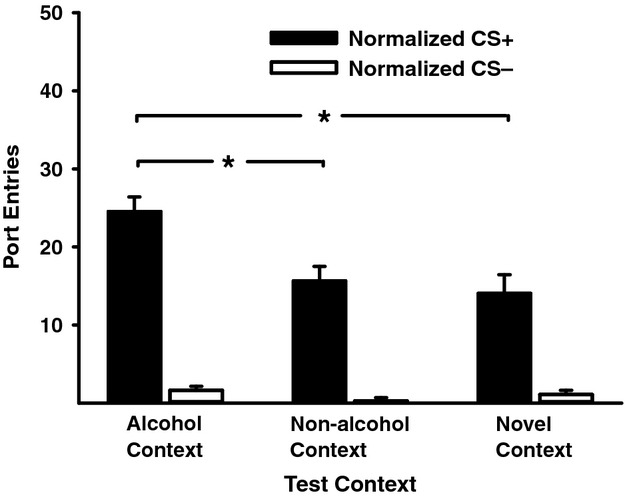
Port entries in response to the alcohol-predictive CS+ are invigorated in an alcohol context, compared to a nonalcohol context or a novel context. Data represent mean (± SEM) normalized port entries during the CS+ (filled bars) and CS− (open bars) at test in each context, where the CS+ and CS− were presented without ethanol. *P < 0.05 for indicated comparisons.

As in Experiment 1, rats learned to discriminate between the alcohol-paired CS+ and the CS− across PDT sessions (data not shown). Following exposure to a nonalcohol context, CS+ responding was tested in the alcohol-associated context, nonalcohol context or novel context. At test, alcohol seeking elicited by the CS+ was more robust in the alcohol-associated context, when compared to the nonalcohol context or the novel context (Fig. 3). ANOVA revealed significant main effects of CS (*F*(1, 25) = 124.88, *P* < 0.001) and Test Context (*F*(2, 50) = 11.04, *P* < 0.001) and a significant Test Context × CS interaction (*F*(2, 50) = 8.55, *P* = 0.001). Follow-up *t*-tests for paired-samples verified that CS+ responding was higher in the alcohol context compared to the nonalcohol context (*t*(25) = 3.61, *P* = 0.001), or the novel context (*t*(25) = 3.93, *P* = 0.01). There was no difference in the level of CS+ responding at test in the nonalcohol context and novel context, *t*(25) = 0.70, *P* = 0.49. Rats made more port entries during the CS− in the alcohol context compared to the nonalcohol context (*t*(25) = 2.24, *P* = 0.03). There was no significant difference in CS− responding in the alcohol context compared to the novel context (*t*(25) = 0.74, *P* = 0.46) or in the nonalcohol context and the novel context (*t*(25) = −1.53, *P* = 0.14). There was no impact of Test Context (*F*(2, 50) = 0.89, *P* = 0.42) on the number of port entries made outside the CS+ (mean ± SEM: alcohol-associated context, 23.96 ± 3.95; nonalcohol context, 19.42 ± 3.29; novel context, 26.27 ± 4.24), suggesting that the alcohol-associated context selectively invigorated CS+ responding.

### Experiment 3: Impact of context extinction on Pavlovian-conditioned alcohol-seeking behavior

As in the previous two experiments, rats learned to discriminate between the alcohol-paired CS+ and the CS− across PDT sessions (data not shown). Following PDT, rats were either exposed to the PDT context (Group 1, context-extinction) or to a different context (Group 2, alternate context) for eight sessions in which neither the cues nor alcohol were presented (see Fig. S1). Subsequently, responding to the CS+ and CS− without alcohol was tested in the context in which PDT had been conducted.

**Figure 4:**
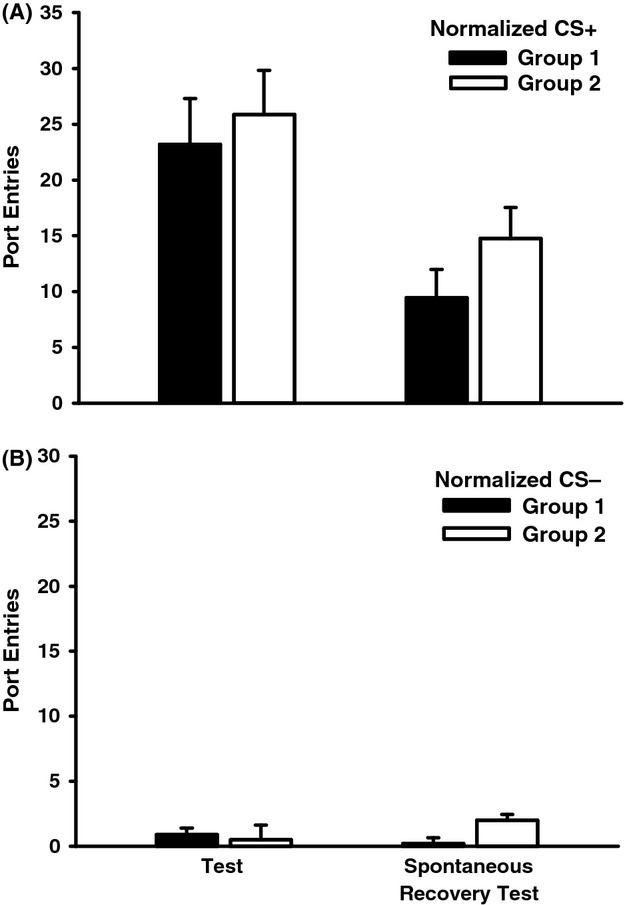
Extinguishing the excitatory properties of the alcohol context did not influence responding to the CS+ or CS− at test. Responding to each CS without ethanol was assessed 24 h after the final session of alternate, nonalcohol context exposure or context-extinction (Test 1) and also 10 days later (Spontaneous Recovery Test). Filled bars represent data from rats that received context extinction (Group 1) and open bars represent data from rats that received alternate-context exposure (Group 2) (A) Mean (± SEM) normalized port entries during the CS+. (B) Mean (± SEM) normalized port entries during the CS−.

There was no impact of context extinction on normalized CS+ (Fig. 4A) or normalized CS− responses (Fig. 4B) averaged over either Test 1 or the spontaneous recovery test. ANOVA conducted on normalized CS responses from Test 1 revealed a significant main effect of CS (*F*(1, 15) = 65.20, *P* < 0.001), but no main effect of Group (*F*(1, 15) = 0.15, *P* = 0.70) or Group × CS interaction (*F*(1, 15) = 0.27, *P* = 0.61). Similar outcomes were obtained at the test for spontaneous recovery (CS, *F*(1, 15) = 31.01, *P* < 0.001; Group, *F*(1, 15) = 3.67, *P* = 0.07; Group × CS interaction, *F*(1, 15) = 0.80, *P* = 0.39).

**Figure 5:**
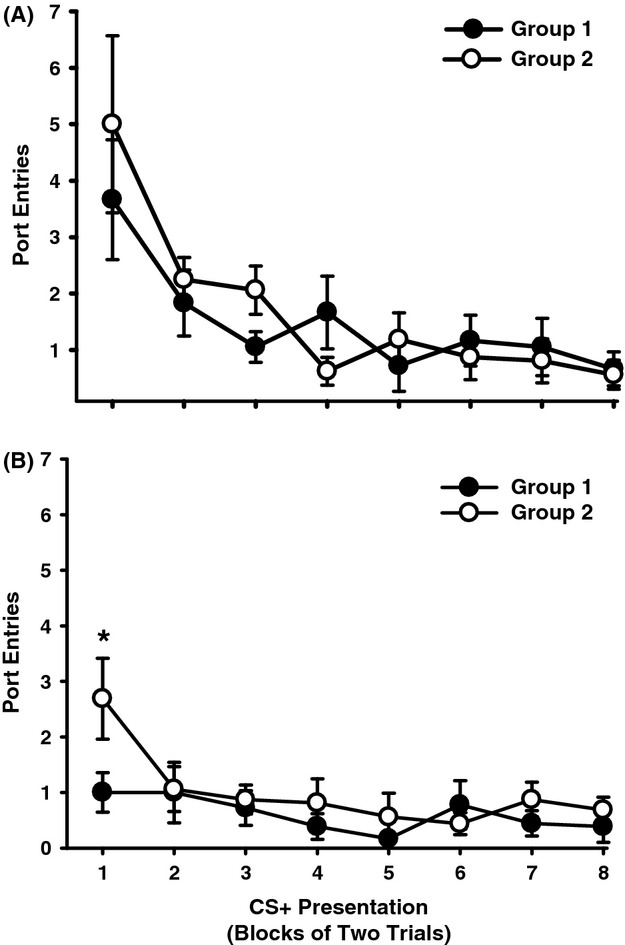
Context extinction reduced responding to the alcohol-predictive CS+ at the start of the spontaneous recovery test. Data represent mean (± SEM) port entries averaged across blocks of two CS+ trials at (A) Test 1 and (B) the spontaneous recovery test. Filled symbols represent data from rats that received context extinction (Group 1) and open symbols represent data from rats that received alternate-context exposure (Group 2). *P < 0.05, Group 1 versus Group 2 at Block 1.

An examination of port entries made during blocks of CS+ trials at test 1 (Fig. 5A) and during the test for spontaneous recovery (Fig. 5B) revealed that rats checked the fluid port more frequently at the start of the session, and that responding decreased across CS+ trials (Test 1, Block, *F*(1, 7) = 7.74, *P* < 0.001; Spontaneous Recovery, Block, *F*(1, 7) = 3.09, *P* = 0.01). There was no main effect of Group (Test 1, *F*(1, 7) = 0.00, *P* = 0.97; Spontaneous Recovery, *F*(1, 7) = 0.92, *P* = 0.35) and no Group × Block interactions (Test 1, *F*(1, 7) = 0.65, *P* = 0.72; Spontaneous Recovery, *F*(1, 7) = 1.13, *P* = 0.35). Because alcohol seeking was highest during initial CS+ trials, *t*-tests for independent samples were used to evaluate group differences at Block 1 to test the specific prediction that an effect of context extinction would only be observed early in the test session. There was no difference between groups at Test 1 (*t*(15) = 0.52, *P* = 0.48). However, during the test for spontaneous recovery rats that had received context extinction made significantly fewer port entries than rats that had received exposure to the alternate context (*t*(15) = 2.17, *P* = 0.05).

**Figure 6:**
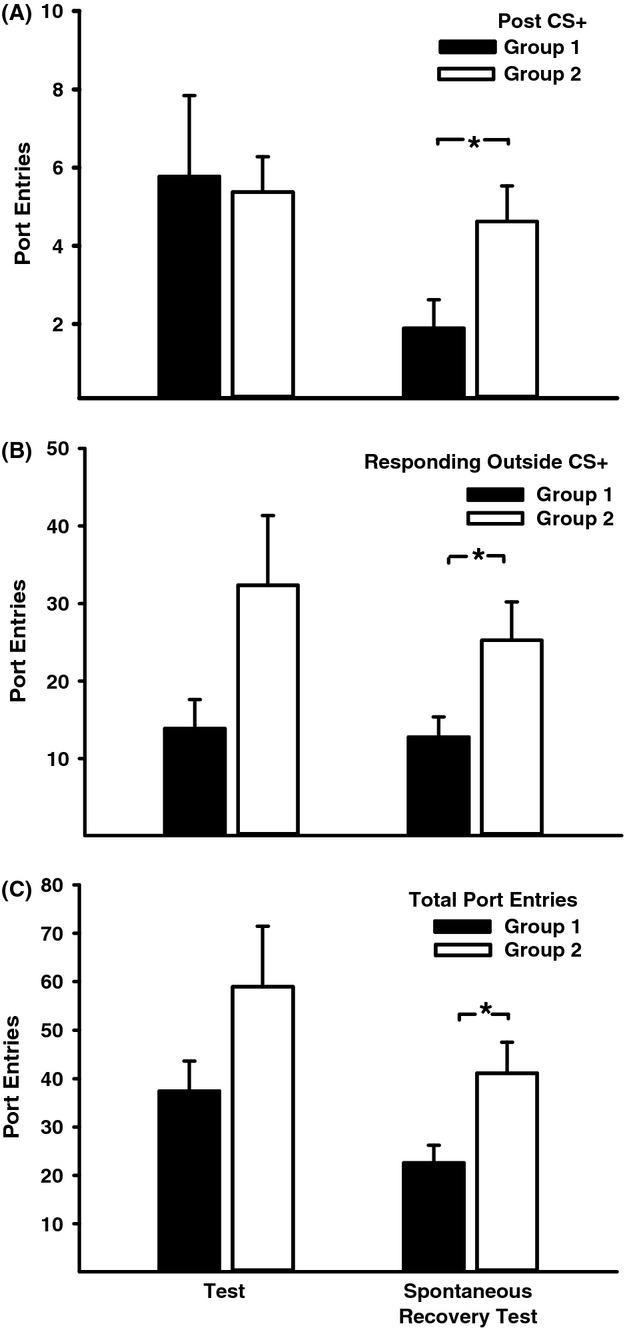
Context extinction reduced context-driven alcohol seeking during the test for spontaneous recovery. Filled bars represent data from rats that received context extinction (Group 1) and open bars represent data from rats that received alternate-context exposure (Group 2) (A) Mean (± SEM) port entries during post-CS+ intervals. (B) Mean (± SEM) port entries that occurred outside CS+ intervals obtained by subtracting port entries during the CS+ from total port entries. (C) Mean (± SEM) total port entries at test. *P < 0.05 for indicated comparisons.

In addition to examining the impact of context extinction on CS+ responding we assessed the effect of this manipulation on alcohol seeking that was not signalled by the CS+ in order to determine if context extinction had an influence on the capacity of the PDT context to directly stimulate alcohol seeking (Fig. 6). ANOVA conducted on port entries made during the 10-sec intervals after each CS+ trial (post-CS+; Fig. 6A) indicated no group differences at Test 1 (Group, *F*(1, 15) = 0.03, *P* = 0.87). However, context extinction significantly reduced post-CS+ responses at the test for spontaneous recovery (Group, *F*(1, 15) = 5.61, *P* = 0.03). Similar results were obtained when comparing group differences in the number of port entries that occurred outside CS+ intervals (Fig. 6B). There was a near significant difference across groups at Test 1 (*F*(1, 15) = 3.94, *P* = 0.07) and a significant reduction in alcohol seeking following context extinction during the spontaneous recovery test (*F*(1, 15) = 5.35, *P* = 0.04). This pattern was again revealed when comparing group differences in total port entries made at each test (Fig. 6C). While groups did not differ at Test 1 (*F*(1, 15) = 2.59, *P* = 0.13), context extinction reduced the total number of port entries made during the test for spontaneous recovery (*F*(1, 15) = 6.70, *P* = 0.02).

## Discussion

The present data reveal that alcohol-seeking behavior triggered by a discrete Pavlovian-conditioned alcohol cue is strongly invigorated by an alcohol-associated environmental context. Alcohol seeking elicited by an alcohol-predictive CS+ was consistently more robust in a context associated with prior alcohol consumption, compared to either novel or familiar contexts in which alcohol had never been consumed. Extinguishing the association between the PDT context and alcohol did not diminish CS+ responding at Test 1, but markedly reduced alcohol-seeking behavior driven by the PDT context during a test for spontaneous recovery. These findings have important implications for craving in individuals with alcohol abuse disorders, as they may encounter discrete and contextual alcohol-predictive cues concurrently.

Rats acquired the predictive relation between the CS+ and alcohol during PDT (Chaudhri et al. 2008b, 2010, 2013). Following training, exposure to a second, alternate context without cues or alcohol was conducted to establish the second context as an environment in which alcohol was never available. Evidence for this association is seen in the across-session decrease in spontaneous entries into the fluid port during this phase (Figs. 2C, S1).

At test, responding to both cues was assessed in several different contexts. It is important to note that cue responding had not been extinguished before Test 1, thereby paralleling human studies that examine craving and physiological reactivity induced by discrete drug cues that have not been systematically extinguished (Staiger and White 1991; Thomas et al. 2005). Consistent with previous data, rats continued to discriminate between the CS+ and CS− when the cues were presented without alcohol in a nonalcohol context (Chaudhri et al. 2010). Discrimination remained intact when the cues were presented in the PDT context where alcohol had previously been consumed: however, alcohol-seeking behavior driven by the CS+ was invigorated in the alcohol-associated context, compared to either the nonalcohol or novel contexts. This effect was consistent across two separate experiments conducted using different concentrations of ethanol during PDT. Thus, the context in which a discrete drug cue is experienced can be a critical determinant of the level of drug seeking elicited by that cue (Zironi et al. 2006; Tsiang and Janak 2006; Chaudhri et al. 2008a; Nees et al. 2012,). When translated to the human condition these results imply that craving may be more vigorous when discrete drug cues are encountered in a drug-associated context, and that the combination of discrete and contextual drug cues may be the more potent trigger for relapse, compared to either type of cue independently.

There was no difference in the level of alcohol seeking driven by the CS+ in either a nonalcohol context or a novel context, indicating that removal from a nonalcohol context per se is not sufficient to invigorate Pavlovian-conditioned alcohol seeking. That the CS+ triggered alcohol seeking in a novel context parallels data from human studies in which reactivity to drug-predictive cues can be evoked in novel laboratory settings that may not resemble environments in which participants normally consume drugs (Litt and Cooney 1999; de Wit 2000; Field and Duka 2002). The present findings suggest that the strength of cue reactivity measured in human studies may be underestimated in laboratory environments. By extension, cue-reactivity estimates might be more accurate if tests could be conducted either in drug-use environments, or in laboratory settings that incorporated contextual elements that might be found in drug-use environments. The use of virtual reality to create drug contexts may prove useful for such investigations (Bordnick et al. 2008; Paris et al. 2011; Traylor et al. 2011).

When presented without alcohol at test, the number of port entries elicited by each CS+ presentation decreased across trials. In Experiment 1, there was no difference in how rapidly this extinction of alcohol seeking occurred as a function of test context. However, CS+ responding was elevated in the alcohol context, compared to the nonalcohol context throughout the test session. This finding suggests that conducting extinction in a context where the unconditioned stimulus, in this case alcohol, was previously experienced produces a resistance to extinction (see also Bouton et al. 2011). This is an important observation given that human addicts may undergo exposure therapy in which drug-predictive discrete cues are repeatedly presented without the drug in an effort to dampen cue reactivity (Drummond and Glautier 1994; Conklin and Tiffany 2002). The rate of extinction during these sessions might be influenced by the setting in which they are conducted, which in turn could impact the longevity of the extinction memory.

Like the present data, studies using instrumental alcohol self-administration procedures also reveal that context can modulate responding to discrete drug cues. In these procedures, subjects are trained to perform an operant response to obtain alcohol, and alcohol delivery is generally paired with a discrete tone-light cue. Following acquisition, responding is extinguished by withholding alcohol. Interestingly, if training and extinction are conducted in distinct contexts then placement into the training context following extinction renews responding. This effect is invigorated by contingent presentations of the discrete tone-light cue at test, compared to tests in which the cue is absent (Tsiang and Janak 2006). Furthermore, when rats are trained to lever-press for alcohol in one context and then extinction is conducted in a second context, exposure to a drop of alcohol triggers reinstatement in the alcohol context but not in the extinction context (Chaudhri et al. 2008a). Congruent with the present data, these results suggest that both instrumental alcohol seeking and Pavlovian-conditioned alcohol-seeking responses can be strongly invigorated by alcohol-associated contexts.

Experiment 3 tested the hypothesis that the facilitation of cue-driven alcohol seeking in the alcohol context is attributable to a summation of the conditioned excitatory properties of the CS+ and the alcohol-associated context. This hypothesis was derived from data showing that drug contexts stimulate craving in humans, suggesting that contexts acquire conditioned excitatory properties (Conklin et al. 2008), and by a preclinical study in rats showing that relative to a neutral context, a context associated with the euphoric effect of morphine facilitated sexual behavior triggered by the presence of a female rat (Mitchell and Stewart 1990). Thus, a context associated with one positive unconditioned stimulus can invigorate responding elicited by a conditioned stimulus that predicts a different positive unconditioned stimulus. We reasoned that if the alcohol context functioned as an excitatory Pavlovian CS, then extinguishing the association between the context and alcohol would result in less responding to the CS+ at test relative to subjects that had not received context extinction. Context extinction has been used as an experimental manipulation to study the influence of contexts on responding to Pavlovian-conditioned cues that predict aversive events (Bouton and Bolles 1979). Consequently, following PDT sessions in Experiment 3 rats were repeatedly exposed to either the PDT context without alcohol (context extinction) or to an alternate, nonalcohol context before test. Spontaneous entries into the fluid port decreased across these sessions (Fig. S1), suggesting an extinction of the context-alcohol association.

Contrary to our predictions, context extinction did not reduce responding during the CS+ (Figs. 4A, 5A) or immediately after the CS+ (Fig. 6A) at Test 1. However, there was a trend for port entries made during intervals of the test session that were not signalled by the CS+ (Fig. 6B) to be reduced at test 1 following context-extinction. The negligible impact of context extinction on CS+ responding at Test 1 suggests that discrete alcohol-predictive cues are highly effective at driving alcohol-seeking behavior. However, the unequal number of PDT and context-extinction sessions may also have contributed to this result, and conducting equivalent numbers of PDT and context-extinction sessions might have revealed an effect of context extinction on CS+ responding.

Interestingly, a marked effect of context extinction was found in a test for spontaneous recovery that was conducted 10 days after Test 1. Context extinction resulted in a modest but significant reduction in CS+ responding at the start of the spontaneous recovery test (Fig. 5B). Moreover, rats that received context extinction made fewer port entries overall (Fig. 6C), particularly during time intervals that were not signalled by the CS+ (Fig. 6B). Thus, extinguishing the excitatory properties of the PDT context appeared to more effectively reduce alcohol-seeking behavior triggered directly by the context, relative to alcohol-seeking responses triggered by the CS+. However, the test for spontaneous recovery was different from Test 1 in that it was the second experience of the CS+ being presented without alcohol in the (extinguished) PDT context. The efficacy of context extinction might therefore have been enhanced by prior extinction of CS+ responding in the PDT context during Test 1.

The reduction in unsignaled alcohol-seeking responses during the test for spontaneous recovery following context extinction suggests that an alcohol-associated context can function as a Pavlovian conditioned stimulus that directly elicits alcohol-seeking behavior. That context extinction had no impact on CS+ responding at Test 1 suggests that the conditioned excitatory properties of discrete and contextual alcohol cues do not summate. Another mechanism by which context can influence responding to discrete cues is by functioning as an occasion-setter, which is a stimulus that modulates the capacity of another stimulus to elicit a response, but does not elicit a response itself (Bouton 2004; Crombag et al. 2008). This property may explain the modest decrease in CS+ responses during the test for spontaneous recovery, in which rats that had previously received context extinction received a CS+ whose association with alcohol may also have been diminished as a result of the CS+ being presented without alcohol during Test 1.

In summary, our results indicate that alcohol-seeking behavior elicited by a discrete alcohol cue is robustly invigorated in an alcohol-associated context. These findings suggest that the strongest trigger for drug craving and potentially relapse in humans might be the combined experience of discrete drug cues in a drug-associated context. Context extinction reduced alcohol-seeking behavior triggered directly by the PDT context, supporting the hypothesis that drug contexts can acquire conditioned excitatory properties through Pavlovian learning. Based on these findings, exposure treatments aimed at diminishing the impact of drug-predictive cues through extinction training in human addicts should consider targeting both discrete and contextual drug-predictive cues.

## Supporting Information

Figure S1 Mean (± SEM) total port entries across sessions in which neither cues nor alcohol were presented. For rats in Group 1 these sessions were conducted in the PDT context (filled symbols) and for rats in Group 2 these sessions were conducted in a distinct, nonalcohol context (open symbols). ANOVA revealed no main effect of Group, *F*(1, 7) = 3.02, *P* = 0.10, and no Group × Session interaction, *F*(1, 7) = 1.15, *P* = 0.34. There was, however, a main effect of Session, *F*(1, 7) = 3.93, *P* = 0.01. Total port entries collapsed across group decreased from an average (mean ± SEM) of 39.06 ± 7.71 on session 1 to 18.88 ± 6.28 (mean ± SEM) on session 8.
