# Supplementary material for: Pavlovian-conditioned alcohol-seeking behavior in rats is invigorated by the interaction between discrete and contextual alcohol cues: implications for relapse
Source: Brain Behav. 2014 Feb 6;4(2):278–89. doi: 10.1002/brb3.216 (PMC3967542; doi:10.1002/brb3.216)
Supplement: Figure S1 — Mean (± SEM) total port entries across sessions in which neither cues nor alcohol were presented. For rats in Group 1 these sessions were conducted in the PDT context (filled symbols) and for rats in Group 2 these sessions were conducted in a distinct, nonalcohol context (open symbols). ANOVA revealed no main effect of Group, F(1, 7) = 3.02, P = 0.10, and no Group × Session interaction, F(1, 7) = 1.15, P = 0.34. There was, however, a main effect of Session, F(1, 7) = 3.93, P = 0.01. Total port entries collapsed across group decreased from an average (mean ± SEM) of 39.06 ± 7.71 on session 1 to 18.88 ± 6.28 (mean ± SEM) on session 8. [file brb30004-0278-sd1.pdf]

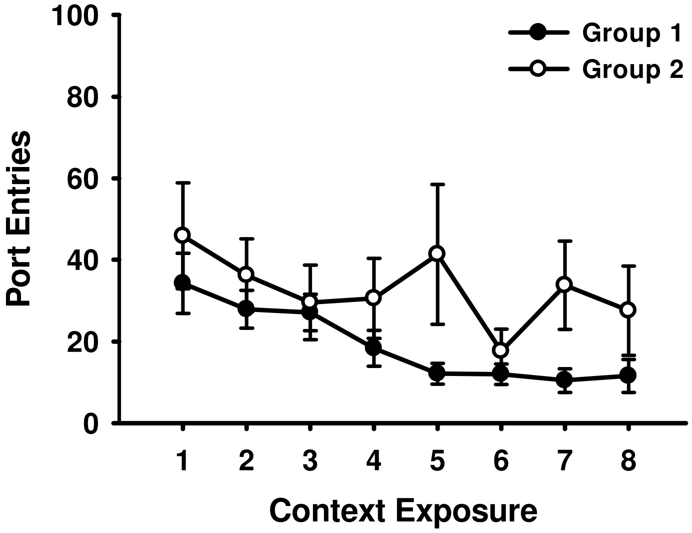


Supplementary Figure 1. Mean (± SEM) total port-entries across sessions in which neither cues nor alcohol were presented. For rats in Group 1 these sessions were conducted in the PDT context (filled symbols) and for rats in Group 2 these sessions were conducted in a distinct, non-alcohol context (open symbols). ANOVA revealed no main effect of Group [$F(1,7)=3.02$, $P=0.10$] and no Group x Session interaction [$F(1,7)=1.15$, $P=0.34$]. There was however, a main effect of Session [$F(1,7)=3.93$, $P=0.01$]. Total port-entries collapsed across group decreased from an average (Mean ± SEM) of 39.06 ± 7.71 on session 1 to 18.88 ± 6.28 (Mean ± SEM) on session 8.
